# Experimental observation of drumhead surface states in SrAs_3_

**DOI:** 10.1038/s41598-020-59200-2

**Published:** 2020-02-17

**Authors:** M. Mofazzel Hosen, Gyanendra Dhakal, Baokai Wang, Narayan Poudel, Klauss Dimitri, Firoza Kabir, Christopher Sims, Sabin Regmi, Krzysztof Gofryk, Dariusz Kaczorowski, Arun Bansil, Madhab Neupane

**Affiliations:** 1 Department of Physics, University of Central Florida, Orlando, Florida 32816 USA; 2 Department of Physics, Northeastern University, Boston, Massachusetts 02115 USA; 3 Idaho National Laboratory, Idaho Falls, Idaho 83415 USA; 4 Institute of Low Temperature and Structure Research, Polish Academy of Sciences, 50-950 Wrocław, Poland; 5 Institute of Molecular Physics, Polish Academy of Sciences, Mariana Smoluchowskiego 17, 60-179 Poznań, Poland

**Keywords:** Materials science, Physics

## Abstract

The topological nodal-line semimetal (TNS) is a unique class of materials with a one dimensional line node accompanied by a nearly dispersionless two-dimensional surface state. However, a direct observation of the so called drumhead surface state within current nodal-line materials is still elusive. Here, using high-resolution angle-resolved photoemission spectroscopy (ARPES) along with first-principles calculations, we report the observation of a topological nodal-loop (TNL) in SrAs_3_, whereas CaAs_3_ exhibits a topologically trivial state. Our data reveal that surface projections of the bulk nodal-points are connected by clear drumhead surface states in SrAs_3_. Furthermore, our magneto-transport and magnetization data clearly suggest the presence (absence) of surface states in SrAs_3_ (CaAs_3_). Notably, the observed topological states in SrAs_3_ are well separated from other bands in the vicinity of the Fermi level. RAs_3_ where R = Ca, Sr, thus, offers a unique opportunity to realize an archetype nodal-loop semimetal and establish a platform for obtaining a deeper understanding of the quantum phase transitions.

## Introduction

Experimental discoveries of non-trivial topological states in semimetals such as the Dirac^1–4^, Weyl^5–8^, and nodal-line^9–12^ semimetals have greatly expanded the family of available topological materials beyond topological insulators^13–18^. In the case of the node line/loop semimetals the valence and conduction bands touch along lines/loops in the Brillouin zone and disperse linearly in directions perpendicular to these lines. The density of states at the Fermi energy in an NLS is greater than that of a Dirac or Weyl semimetal, providing a more favorable condition for investigating exotic non-trivial phases and realistic material platforms for developing applications. Note that, the NLSs are not robust against spin-orbit coupling or other perturbations and require crystal symmetries for their protection. To date, several structural classes of NLSs such as PbTaSe_2_^19^, LaN^20^, Cu_3_PdN^21^, and ZrSiS-type^10,11,22–27^ materials have been reported with associated space group symmetries that protect the nodal-line state. However, the nodal-loop states in PbTaSe_2_^19^, and Cu_3_PdN^21^ lie in the vicinity of other metallic bands, LaN requires multiple symmetries for protection, while in the ZrSiX-type systems the topological states lie above the Fermi level. It is highly desirable, therefore, to find materials which require minimum symmetry protections without the presence of other nearby bands that interfere in isolating topological features within the electronic spectrum.

It has been recently shown that time-reversal symmetry (TRS) with a center of inversion symmetry (CIS) is sufficient, in principle, to protect a nodal-line state^28–30^. APn_3_ (A = Ca, Sr, Ba, Eu; Pn = P, As) family of compounds has been identified as a potential material class to host such a minimal symmetry protected NLS when SOC is excluded^29,30^. Among these, CaP_3_ and CaAs_3_ are the only members of this series to have a triclinic crystal structure with space group *P1*, whereas other members including SrAs_3_ crystallize with higher symmetry structures characterized by space group *C2/m*. Remarkably, in the *P1* space group, CIS is the only crystalline symmetry that can protect the topological nodal-line states along with TRS^30^. So that, such a system can aptly work as the material platform of an ideal nodal-loop system. However, the experimental verification of this tempting conjecture has not been reported yet. Our studied material RAs_3_ (R = Ca, Sr) could provide not only the nodal-loop state but also the topological surface state or drumhead surface states in momentum space connecting nodal points. RAs_3_ thus appears to be a system with an enhanced topological density of states at the Fermi surface, paving the road for the potential discovery of more exotic states.

Here, we report the experimental observation of a topological nodal-loop state in the monoclinic system SrAs_3_ and a trivial state in the triclinic system CaAs_3_ in its (010) surface. Utilizing angle-resolved photoemission spectroscopy (ARPES), we systematically study the detailed electronic structure of these materials. Our ARPES data and first-principles calculations reveal the presence of a topological nodal-loop state around the center (Y) of the Brillouin zone (BZ) in SrAs_3_. Furthermore, we observe a drumhead surface state connecting the nodal-point projection along the Y direction. Our magnetotransport data show clear signatures of quantum oscillations suggesting the presence of surface states in SrAs_3_, while CaAs_3_ lacks such oscillations in magnetic fields up to 9 T. Our experimental data are corroborated by our first-principles calculations. Interestingly, our calculations suggest that CaAs_3_ undergoes a topological phase transition from TNL to TI when SOC is turned on (also see refs. ^29,30^). Furthermore, our experimental data reveal that the Fermi surface of CaAs_3_ is formed by a sole band. Therefore, our study could open up a new platform for studying the interplay between various topological phases.

## Results

### Crystal structure and sample characterizations

**Figure 1:**
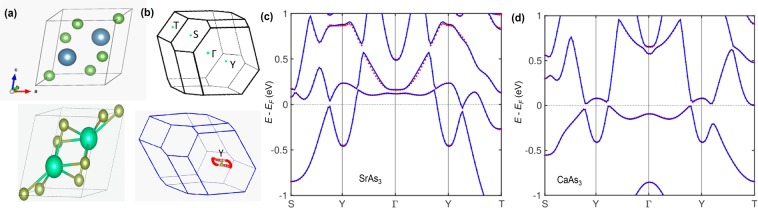
Crystal structure and sample characterization of RAs_3_. (**a**) Triclinic (upper panel) and monoclinic (lower panel) primitive unit cell. Purple (neon green) and green balls represent Ca(Sr) and As atoms, respectively. The center of inversion lies between the two neighboring Ca(Sr) atoms. (**b**) 3D Brillouin zone of RAs_3_ with the high symmetry points (upper panel) are marked. The nodal-line is located around the Y points. A little deviation from the S-Y-T plane (lower panel) is seen here for CaAs_3_. (**c**,**d**) Bulk band structure along the high symmetry points calculated with the inclusion of SOC for SrAs_3_ and CaAs_3_, respectively. Blue lines and dots correspond to the tight-binding model and first-principles calculations, respectively.

The triclinic crystal structure of CaAs_3_ is shown in Fig. 1(a) (upper panel). The center of inversion lies midway between the neighboring Ca atoms. The crystal structure of SrAs_3_ has higher symmetry compared to that of CaAs_3_ (see Fig. 1(a)), hence, SrAs_3_ crystallizes in a simple monoclinic structure with space group *C2/m*. Therefore, in addition to the center of inversion symmetry, SrAs_3_ has *C2* rotational symmetry. The center of inversion symmetry lies midway between two Sr atoms and the two-fold rotational symmetry can be readily observed from the primitive monoclinic unit cell as shown in Fig. 1(a) (lower panel). The corresponding bulk Brillouin zone with high symmetry points is shown in the upper panel of Fig. 1(b). An important point to note that the projections of Y and Γ points on the (010) plane are located at the same point of the BZ. Moreover, note that for the (010) hexagonal surface (around Y), one axis is larger than the other two axes. The lower panel of Fig. 1(b) demonstrates the location of a nodal-loop centered around the Y point while the spin-orbit coupling (SOC) effect is excluded; here a little deviation from the S-Y-T plane is observed for CaAs_3_ (note inplane for SrAs_3_).

Figure 1(c,d) show the bulk electronic band structure of CaAs_3_ and SrAs_3_, respectively, calculated along the various high symmetry directions using tight binding (lines) and first-principles (dots) techniques considering the spin-orbit coupling (SOC) effect. Analyzing the calculations of both materials without SOC, one finds a nodal-loop around the Y point of BZ, which is located in the vicinity of the chemical potential (see Supplementary Fig. 2 in see Supplementary Information for additional data and related analysis). An important point to note that the bands are fully gapped as they diverge from the Y points in both directions. The small gap in CaAs_3_ is due to the fact that the nodal points lie slightly away from the high symmetry points. The inclusion of SOC results in opening a negligible gap in SrAs_3_ and an approximately 40 meV gap in CaAs_3_ along the Y-Γ direction (see Supplementary Information for additional data and related analysis for details of both with and without SOC calculations). The observed instability of the nodal-loop phase against the fully-gapped topological phase is in concert with the experimental electrical resistivity data of CaAs_3_, where a crossover from semimetallic to low-temperature insulating behavior occurs. However, the insulating character is sufficiently weak enough to neglect in SrAr_3_ for both the transport measurements and first-principles calculations (see Supplementary Information for additional data and related analysis). Importantly, the exclusion of SOC to observe nodal-line or loop states is a well-known prevalent technique that has played a significant role in realizing previously reported nodal-line semimetals such as LaN^20^, Cu_3_(Pd,Zn)N^21^, ZrSiX-type materials^10,11,25^, etc.

### Fermi surface and constant energy contour plots of RAs_3_

**Figure 2:**
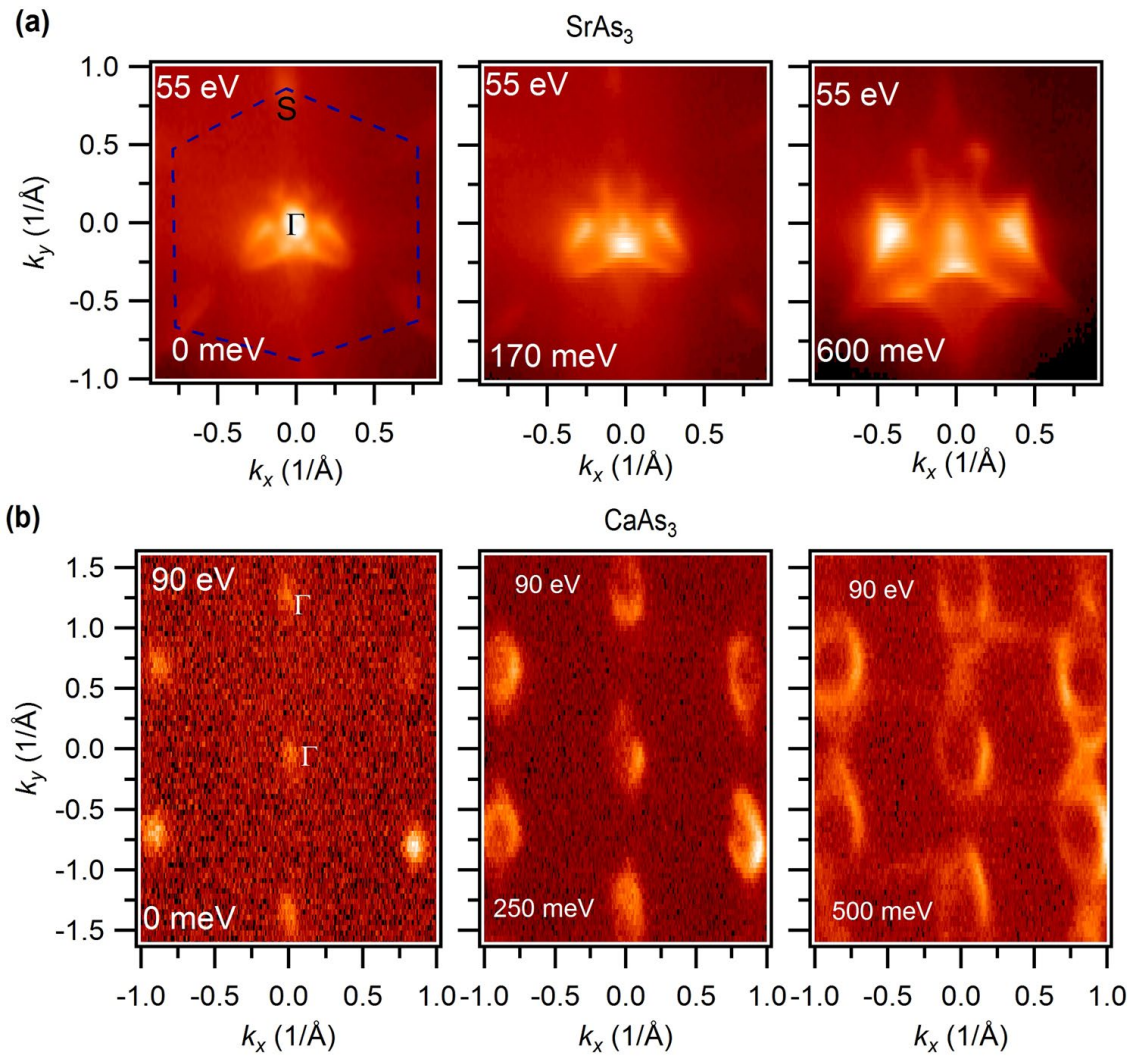
Fermi surface and constant energy contours of RAs_3_ (**a**) Fermi surface and constant energy contour plots of SrAs_3_, measured at the ALS beamline 10.0.1 using a photon energy of 55 eV. (**b**) Measured Fermi surface and constant energy contour plots of CaAs_3_. Each of the distorted hexagons represents a separate Brillouin zone where the b-axis is larger than the a-axis. The measurements were performed at the HRPES end-station of the SLS beamline at a temperature of about 18 K using an incident photon energy of 90 eV. The binding energies are marked in the plots.

**Figure 3:**
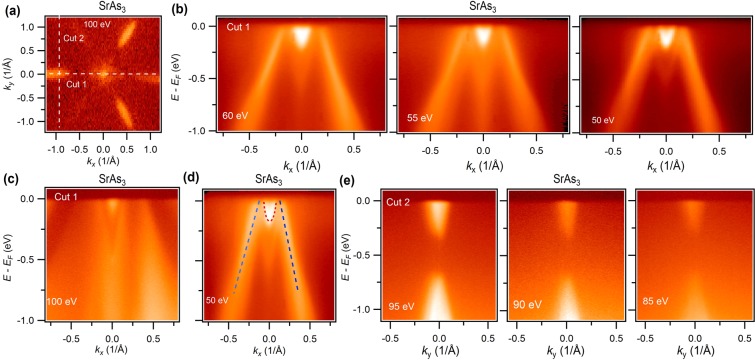
Observation of nodal-loop state in SrAs_3_. (**a**) Fermi surface map at a photon energy of 100 eV. (**b**) Photon energy dependent dispersion maps along the cut 1 direction. (**c**) Dispersion map measured at 100 eV photon energy. (**d)** Dispersion map with guide to the eyes. The red dotted arc shows the drumhead surface state. (**e)** Dispersion map along the center of the electron like pocket. Experimental data (**a**,**c**,**e**) were taken at the SLS and (**b**) and (**d**) were taken at the ALS beamline 10.0.1 at a temperature around 18 K. The photon energies are marked in the plot.

In order to determine the nature of the charge carriers and to unveil the Fermi surface evolution with the binding energy, we present the Fermi surface and constant energy contour plots in Fig. 2 for the (010) surface. The hexagonal Fermi surface (blue dashed line) of SrAs_3_ is observed with 55 eV incident photon energy in Fig. 2(a). As discussed earlier for the (010) surface, our measured Fermi surface Γ refers to Y point. At the center, we clearly observe a circular pocket which is a result of the surface arc-like state near the Fermi level, namely the drumhead surface state. Furthermore, we observe six petal-like pockets resembling a flower like shape. Moving towards the higher binding energy (170 meV), we observe that the circular pocket almost disappears and the six petals begin to overlap each other creating a complex feature. The oval shape at the corner also evolves into a small point-like shape. At around 600 meV below the chemical potential, the oval shape and the circular pocket at the zone center completely disappear indicating the electron-like nature of the bands around these points. However, the six flower petal-shaped features evolve into a complex flying bat like feature and confirms the hole-like nature of these bands. From the bulk band calculations, one can easily speculate that the six petals will form a bigger nodal ring around the drumhead surface state. However, the corner of the Brillouin zone is not well resolved at this photon energy. Therefore, we conduct a Fermi surface mapping at a higher incident energy (100 eV) at the SLS beamline which further confirm the hexagonal nature of the Brillouin zone (BZ) (see Fig. 3(a) and also see Supplementary Information for additional data and related analysis). Figure 2(b) (left) shows the experimental Fermi surface map of CaAs_3_ within a wide momentum window. Unlike SrAs_3_, we do not observe the electron-like pockets and flower petal shape at the corner and center of the BZ. Each of the hexagons observed represents an individual BZ of CaAs_3_. In order to figure out the evolution of the Fermi surface contour, we present the constant energy contour plots in Fig. 2(b) (right) and in see Supplementary Information for additional data and related analysis. In these figures, one can clearly see the distorted hexagonal shape of the BZ and hole-like nature of the carriers, that is perfectly reproduced by our calculations (see Supplementary Information for additional data and related analysis).

### Observation of nodal-loop state in SrAs_3_

**Figure 4:**
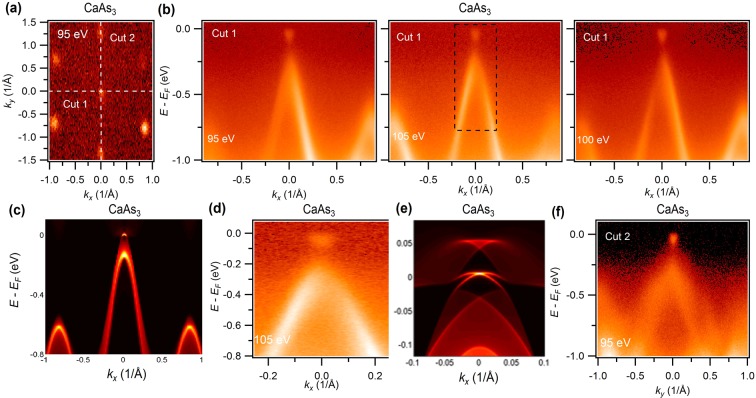
Dispersion maps along the high symmetry directions in CaAs_3_. (**a**) Fermi surface map at a photon energy of 95 eV. White dashed line guides the energy-momentum dispersion measurement directions. (**b**) Photon energy dependent dispersion maps along the cut 1 direction shown in Fig. 4(a). (**c**) Calculated dispersion map without the inclusion of SOC around the Y point of BZ. At the (010) surface, Y and Γ are projected at the same point. (**d**) Zoomed in plot of black dashed box shown in 105 eV dispersion map. (**e**) Calculated zoomed-in plot near the Fermi level. (**f**) Measured dispersion map along the cut 2 direction at a photon energy of 95 eV.

In order to determine the nature of the electronic bands associated with the nodal-loop near the Fermi level, the photon energy dependent energy-momentum dispersion maps are measured (see Figs. 3 and 4 and see Supplementary Information for additional data and related analysis). Figure 3(a) shows the Fermi surface map measured at 100 eV photon energy. The white dashed lines represent the cut directions for the energy-momentum dispersion measurements. Figure 3(b) shows the photon energy dependent dispersion maps along the cut 1 direction of SrAs_3_. Here, we observe the 2D Fermi surface states which correspond to the drumhead surface states at the Γ-point for all the photon energies. The bulk bands below the surface states are not well resolved at the low photon energies, therefore we plot the dispersion map at 100 eV (see Fig. 3(c)). Here, one can clearly observe the bulk bands which provide an explanation for the flying bat like shape in the BZ at higher binding energies. Furthermore, the Dirac point of the nodal-loop and the arc along the Y-Γ direction meet in the vicinity of the Fermi level. Most importantly, the arc-like state does not show any notable dichotomy with photon energies, hence, we conclude that it is surface originated (also see Fig. 3(d) for eye guides and see Supplementary Information for additional data and related analysis). This further confirms our observation of the drumhead surface states and the nodal-loop state which is in agreement with our first-principles calculations (see Fig. 1(d) and refs. ^29,30^). Next, we present the dispersion maps along the six electron pockets observed at the corner in Fig. 3(e) (cut 2 direction). A massive Dirac like state is observed with a ~0.3 eV gap size.

### Observation of trivial elctronic structure in CaAs_3_

Figure 4 represents the dispersion maps along the various high-symmetry directions of CaAs_3_ (see Fig. 4(a)). We used several photon energies for probing different values of the perpendicular components of the crystal momentum. From the results presented in Fig. 4(b) (see also see Supplementary Information for additional data and related analysis), it is clear that only a single band appears in the vicinity of the chemical potential without any interference from irrelevant bands. Our calculations suggest that the surface states lie within the upper part of the band, which is located slightly below the chemical potential. Such a naturally tuned clean system in the vicinity of the chemical potential is very crucial for transport behavior as well as for applications. To understand the nature of the bands along this high symmetry direction in the (010) plane, we carried out the band-dispersion calculations without (see Fig. 4(c)) and with (see Supplementary Information for additional data and related analysis) the inclusion of SOC. The nodal-loop (without SOC) and the surface state (with SOC) are found around the Y point along the ${k}_{x}$ momentum plane. Note that ${k}_{x}$ and ${k}_{y}$ are defined here along the x and y directions and are not defined along the vector direction shown in SF. 2(b) (see Supplementary Information for additional data and related analysis). Most interestingly, the projections of the nodal points in the ${k}_{x}$ direction are connected by the surface states. However, the inclusion of SOC opens up a 40 meV gap along this direction and the system undergoes a topological phase transition from TNL to TI. However, a careful photon energy dependent dispersion map study along the expected nodal line direction reveals a small gap (see Fig. 4(b)) in the vicinity of the nodal loop, which nicely agrees with our first-principles calculations. To closely look at the surface state in CaAs_3_, we show a zoomed-in view of the experimental dispersion map, and results of calculations performed near the chemical potential by including SOC (see Fig. 4(d,e)). Interestingly, in Fig. 4(d), as expected from theoretical calculations, we do not see the surface state within the top part near the Fermi level but a finite gap is observed. From the photon-energy-dependent measurements (see SF.5), one can clearly see the bulk nature of the bands. Importantly, the band around Y shows a sharp *k*_*z*_-dependency and the upper part of the band completely vanishes above the Fermi level at 80 eV dispersion map (similarly at 110 eV), which indicates the 3D nature of the bands. The presence of the surface state, on the other hand,-can be expected at any photon energy, therefore the observation of pure bulk bands negates the possible presence of a Dirac cone with a surface arc in CaAs_3_. We conclude that our experimentally observed state in CaAs_3_ is topologically trivial in nature. With no other bands near the Fermi level, CaAs_3_ thus provides a unique opportunity to see the evolution from the TNL phase to the TI phase through small doping. Figure 4(e) shows the calculated dispersion map near the Fermi level where one can see the nearly flat surface state connecting the bulk bands. Figure 4(f) represents the measured dispersion map along the *k*_*y*_ directions which clearly supports our previous observations. Here, we observe that the band is almost flattened in the *k*_*y*_ direction while we find a sharp dispersion along the *k*_*x*_ direction. This could further provide a tuning knob to study more exciting exotic states.

### Transport and magnetic measurements on SrAs_3_ and CaAs_3_

**Figure 5:**
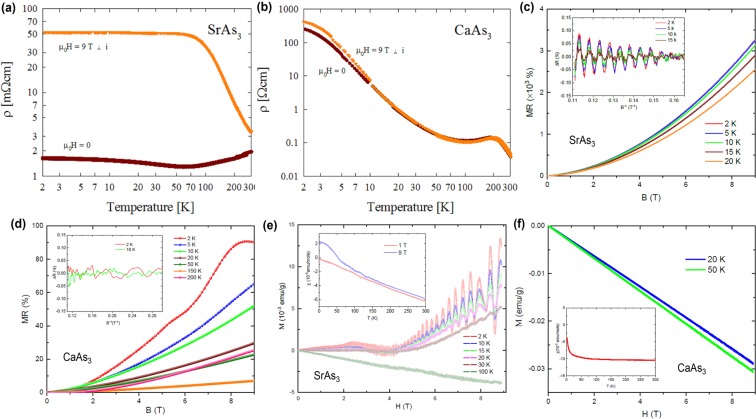
Observation of quantum oscillations of RAs_3_. (**a,b**) Temperature dependencies of the electrical resistivity (note double-logarithmic scales) of SrAs_3_ and CaAs_3_, respectively, measured in zero magnetic field and magnetic field of 9 T applied perpendicular to electric current. (**c,d**) Transverse magnetoresistance of SrAs_3_ and CaAs_3_ measured at different temperatures. The inset shows the SdH oscillations in the case of SrAs_3_ (after subtracting the background from the magnetoresistance data) and lack of the oscillations for CaAs_3_. (**e,f**) Magnetic field dependence of magnetization of SrAs_3_ and CaAs_3_. In case of SrAs_3_ a pronounced dHvA oscillation can be observed while CaAs_3_ shows typical behavior for diamagnetic insulators with no sign of quantum oscillations. The insets show the temperature dependence of magnetic susceptibility of SrAs_3_ and CaAs_3_, respectively.

In order to look in more detail on the electronic behavior and its impact on transport properties, we have performed the electrical resistivity and magnetoresistivity measurements of SrAs_3_ and CaAs_3_ single crystals. The electrical transport behavior of SrAs_3_ is presented in Fig. 5(a). In zero magnetic field, the compound exhibits semimetallic properties with a weak temperature dependent resistivity of about 1.5 mΩcm and a shallow minimum in $\rho$(T) near 60 K, in concert with the literature data^31,32^. In a magnetic field of 9 T, applied perpendicular to the electric current, the resistivity of SrAs_3_ notably changes. In the region from room temperature down to about 70 K, the compound shows semiconducting-like behavior, while at lower temperatures, a plateau in $\rho$(T) is observed, at which the resistivity is ~50 mΩcm, i.e. it is 3000% larger than the magnitude in zero field. Such a distinct influence of the magnetic field on the electrical transport in SrAs_3_ and the presence of the low temperature plateau are characteristic of topological semimetals^33^. This behavior can be attributed to field-induced changes in mobilities and concentrations of electron and hole carriers in a two-band topological material and similar picture was invoked before to explain unusual galvanomagnetic properties of SrAs_3_, like first-order longitudinal Hall effect and magnetoresistivity in Hall geometry^34^. This behavior can support the presence of drumhead surface states in SrAs_3_ as observed in ARPES measurements where the trajectories of the electrons in 2D surface states are easily influenced by magnetic field. A completely different behavior is observed for CaAs_3_ crystals, where the electrical transport measurements show a semiconducting behavior (see Fig. 5(b)). At room temperature, the resistivity is about 37 mΩcm, and with decreasing temperature it increases non-monotonically, initially in a semimetallic manner, passing through a smeared shallow maximum near 200 K, but then rises sharply below 15 K. The resistivity measured at 2 K is about 260 Ωcm, which is a value nearly four orders of magnitude larger than that at 300 K. The overall shape of $\rho (T)$ as well as the values of the resistivity are very similar to those reported in the literature^31,35^. The semiconducting behavior observed in CaAs_3_ agrees with the presence of a small gap close to the Fermi level that has been found by the photoemission studies. The opening of the finite gap and evidence of 3D nature of bands avoids the possibility of Dirac cones, which is consistent with our transport data. As can be inferred from Fig. 5(b), an external magnetic field of 9 T, applied perpendicular to the electric current, hardly affects $\rho$(T) of CaAs_3_ above 10 K, yet brings about a more rapid rise of the resistivity at lower temperatures. The latter feature can be attributed to a small increase in the value of the semiconducting energy gap or/and some reduction in the mobility of dominant charge carriers, both effects being driven by the magnetic field.

Figure 5(c) shows the transverse magnetoresistance (MR) of SrAs_3_ measured at different temperatures and a magnetic field up to 9 T. The MR is defined as the change of the electrical resistance under applied magnetic field and can be described by the formula MR = [R(H) − R(0)]/R(0), where R(H) and R(0) stand for resistance with and without magnetic field, respectively. As can be seen, in the case of SrAs_3_, the MR is positive and non-saturating up to 9 T for all temperatures measured. At low temperatures, the MR reaches large values exceeding 3200% at 2 K and 9 T. Such a large MR in SrAs_3_ may indicate the presence of surface states in this material^36^. The overall MR curve at 2 K can be described by the relation MR ∝ H^*n*^ with n = 1.82. The nearly quadratic field dependence indicates that the system exhibits an almost complete electron-hole compensation, as expected from a semiclassical two band model^36^. At low temperatures and high magnetic field, a signatures of Shubnikov de Haas (SdH) oscillations can be observed. The inset of Fig. 5(c) shows the oscillations versus inverse magnetic field above 6 T at different temperatures and after subtracting the smooth background from the resistivity data (MR ∝ H^1.82^). The results have been normalized at 9 T to better show the magnitude of resistance change caused by the quantum oscillations. The presence of the quantum oscillations, even at 15 K (and 9 T) not only points to a very good quality of the SrAs_3_ single crystals used in the present studies but also indicates the high mobility and extremely low effective mass of charge carriers supporting the presence of surface states in this material. In contrast to SrAs_3_, CaAs_3_ shows a relatively small MR. The MR measured at different temperatures below room temperature are shown in Fig. 5(d). At 2 K, the MR reaches a maximum of 90% at a magnetic field of 8 T and then starts to saturate. At higher temperatures, as shown in Fig. 5(d), the MR is decreasing with increasing temperature and the MR values are non-saturating up to 9 T. Unlike in SrAs_3_, we do not observe any sign of the SdH oscillations in CaAs_3_. The inset of Fig. 5(d) shows the residuals at different temperatures after subtracting the background from resistivity data. As it can be seen, no oscillations are present in the magnetic fields of up to 9 T. This clearly shows a difference between these two systems; SrAs_3_ being a topological semimetal and CaAs_3_ being a trivial semiconductor with a narrow gap in the electronic structure, all in good agreement with the photoemission results. The insulating behavior at low temperature and the relatively small and saturating MR at low temperature could be the reason that both SdH and dHvA oscillations are missing in CaAs_3_. This is in agreement with the ARPES results suggesting that CaAs_3_ is a trivial insulator. Figure 5(e,f) show the field dependences of the magnetization of SrAs_3_ and CaAs_3_, respectively, measured at various temperatures. As it can be observed from the figures for CaAs_3_, the linear field dependence measured is a characteristic feature of a typical diamagnetic insulator. In the case of SrAs_3_ a much complex M(H) behavior is observed with a crossover from weak diamagnetic at weak magnetic field to paramagnetic like behavior at ~5 T. In addition, an obvious de Haas van Alphen (dHvA) oscillations are present for SrAs_3_ at low temperatures and high magnetic fields, as were predicted for topological line node semimetals^37,38^. In nodal line systems, the magnetic susceptibility is composed into the orbital, spin, and spin-orbit cross terms, which is caused by the strong spin orbit interactions^37,38^. In nodal semimetals the spin-orbit cross term is directly related to the chiral surface current (and orbital magnetization) induced by the topological surface modes^37^. In the case of CaAs_3_, no such effects exist (see Fig. 5f) and the magnetization shows an ordinary diamagnetic behavior expected for trivial insulators. The temperature dependence of the magnetic susceptibility of SrAs_3_ and CaAs_3_ is shown in the insets of Fig. 5(e,f), respectively. Whilst CaAs_3_ exhibits a typical behavior expected for an ordinary diamagnetic insulator, the magnetic susceptibility of SrAs_3_ shows an unusual T-linear dependence that may be a signature of its topological nature.

## Discussions

Although few topological nodal semimetals have been realized experimentally, there is a lack of available pristine model systems hosting a well isolated drumhead surface state. Our systematic spectroscopic study reveals the clear signature of the drumhead surface state in SrAs_3_, which is further supported by transport measurements as well as the first-principle calculations. Similarly, our results show that CaAs_3_ is a topologically trivial material with a clear band gap. Therefore, by appropriate isoelectric doping in CaAs_3_ with Sr, the quantum phase transition from topological nodal line phase to the topological insulator state can be realized in Sr_*x*_Ca_1−*x*_As_3_ system. As the topological nodal states are expected to locate in the vicinity of the Fermi level, Sr_*x*_Ca_1−*x*_As_3_ system could provide an ideal platform for transport as well as optical measurements to reveal the topological nodal signatures. By the application of circularly polarized light, one can drive the nodal line phase into a Weyl phase in this system^39^. Our systematic spectroscopic and transport measurements as well as first-principles calculations show that differences in crystal structures, crystallographic symmetry protections, and the SOC strength will lead to substantial differences in the electronic structures.

## Methods

### Sample growth and characterizations

Single crystals of RAs_3_ were grown by Sn-self flux technique as described elsewhere^40^. Chemical composition of the single crystals was checked by energy-dispersive X-ray analysis using a FEI scanning electron microscope equipped with an EDAX Genesis XM4 spectrometer. The average elemental ratios Ca: As and Sr: As obtained in accord with the expected stoichiometry. The crystal structure of the single crystals was examined by X-ray diffraction on a KUMA Diffraction KM-4 four-circle diffractometer equipped with a CCD camera, using graphite-monochromatized Mo-K*α* radiation. The triclinic CaP_3_-type crystal structure of CaAs_3_(space group *P1*, Wyckoff No. 2) and the monoclinic crystal structure of SrAs_3_ (*C2/m*, #12) were confirmed, with the crystal lattice parameters close to the literature values reported in refs. ^35,41^. Measurements of the electrical resistivity were carried out in the temperature range from 2 to 300 K in magnetic field up to 9 T employing a Quantum Design PPMS-9 platform. Electrical contacts were made of silver wires attached to the rectangular-prism-shaped samples with silver epoxy. Because of the very low crystallographic symmetry no effort was made to determine the direction of the electric current in respect to the unit cell axes. The electrical resistivity and magneto-transport properties were measured using standard four-probe technique and magnetic properties were measured using VSM option in PPMS Dynacool-9 (Quantum Design) device.

### Synchrotron measurements

We performed synchrotron-based ARPES measurements at the surface and interface spectroscopy beamline end-station high-resolution photoemission spectroscopy (SIS-HRPES) located at the Swiss Light Source (SLS) which is equipped with Scienta R4000 hemispherical electron analyzer. Similarly we collected more data at the Advanced Light Source (ALS) beamlines 10.0.1.1 and 4.0.3 which are equipped with Scienta R4000 and R8000 hemispherical electron analyzers, respectively. During the data collection, energy and momentum resolution were set better than 20 meV and 0.2°, respectively. We cleaved the samples in ultra high vacuum (UHV) conditions where pressure were better than 10^−10^ torr. The measurement temperatures were set to be 10–25 K. We did not observed any sign of sample degradation during the measurements.

### Theoretical calculations

In order to analyze and interpret the experimental ARPES data, first-principles calculations were performed using both the DFT and TB methods. The DFT calculations were made using Vienna ab-initio simulation package based on Perdew-Burker-Ernzerhof(PBE)^42^ type generalized gradient approximation and the projector augmented-wave(PAW)^43^ pseudopotential. The energy cut-off of 400 eV and a 9 × 9 × 9 k mesh were used to calculate the bulk band structure. A real space TB model based on the Wannier function of As *p* orbitals was built by using WANNIER90^44^ package. The TB model and Green’s function^45,46^ method were employed to calculate the surface band structure and the Fermi surface energy contours.

## Supplementary information

Supplementary Information.

## Data Availability

The data that support the findings of this study are available from the corresponding author upon request.
