# Supplementary material for: Experimental observation of drumhead surface states in SrAs3
Source: Sci Rep. 2020 Feb 17;10:2776. doi: 10.1038/s41598-020-59200-2 (PMC7026427; doi:10.1038/s41598-020-59200-2)
Supplement: Supplementary file 1 — Supplementary Information. [file 41598_2020_59200_MOESM1_ESM.pdf]

Supplementary Information for
Experimental observation of drumhead surface states in $SrAs_3$

M. Mofazzel Hosen,[1] Gyanendra Dhakal,[1] Baokai Wang,[2] Narayan Poudel,[3] Klauss Dimitri,[1] Firoza Kabir,[1] Christopher Sims,[1] Sabin Regmi,[1] Krzysztof Gofryk,[3] Dariusz Kaczorowski,[4] Arun Bansil,[2] and Madhab Neupane*[1]

[1]*Department of Physics, University of Central Florida, Orlando, Florida 32816, USA*

[2]*Department of Physics, Northeastern University, Boston, Massachusetts 02115, USA*

[3]*Idaho National Laboratory, Idaho Falls, Idaho 83415, USA*

[4]*Institute of Low Temperature and Structure Research,*
Polish Academy of Sciences, 50-950 Wrocław, Poland

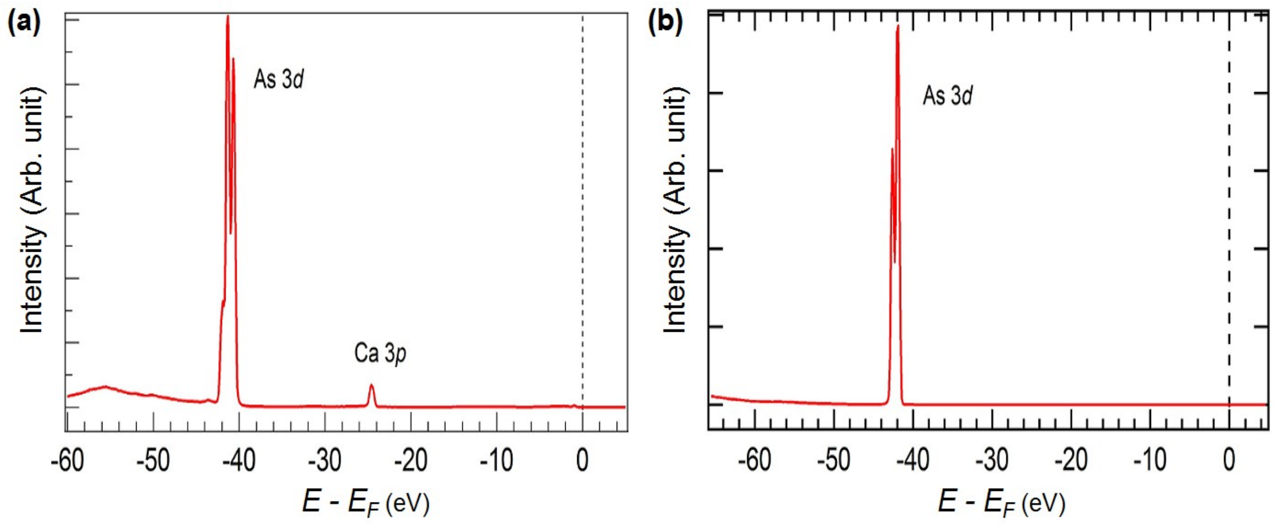

Supplementary figure 1: **Spectroscopic core level measurements.** (a),(b) Core level measurements of $CaAs_3$ and $SrAs_3$, respectively.

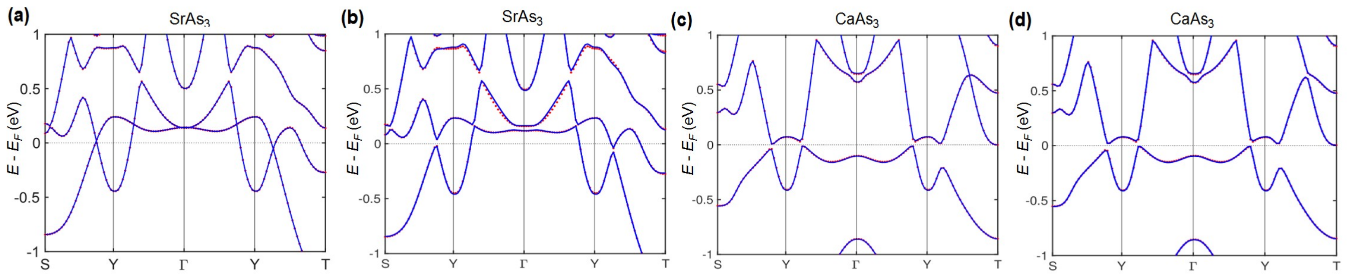

Supplementary figure 2: **First-principles calculations.** (a)-(b) Bulk band calculations without and with spin-orbit coupling (SOC) of $SrAs_3$, respectively. (c)-(d) Calculated bulk bands of $CaAs_3$ along the high symmetry directions without and with SOC, respectively.

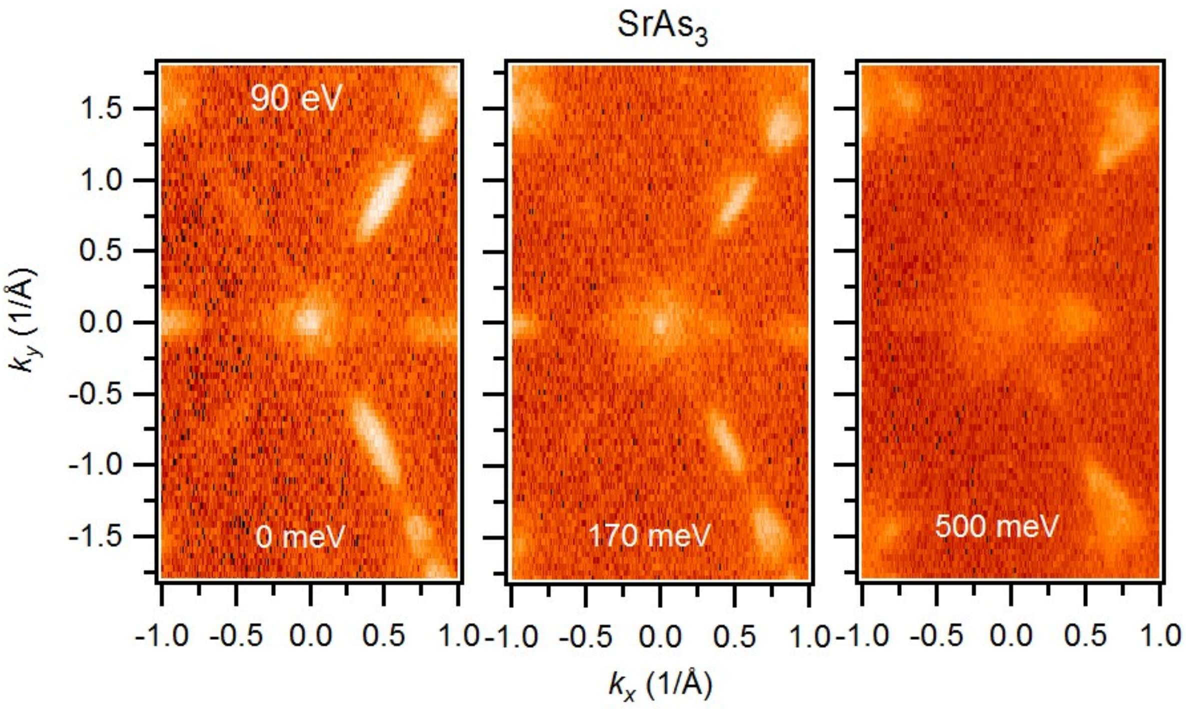

Supplementary figure 3: **Fermi surface of $\mathbf{SrAs_3}$.** Fermi surface (left) and constant energy contour plots of $SrAs_3$ measured at a photon energy of 90 eV. The measurements were performed at the SLS beamline at a temperature of 18 K.

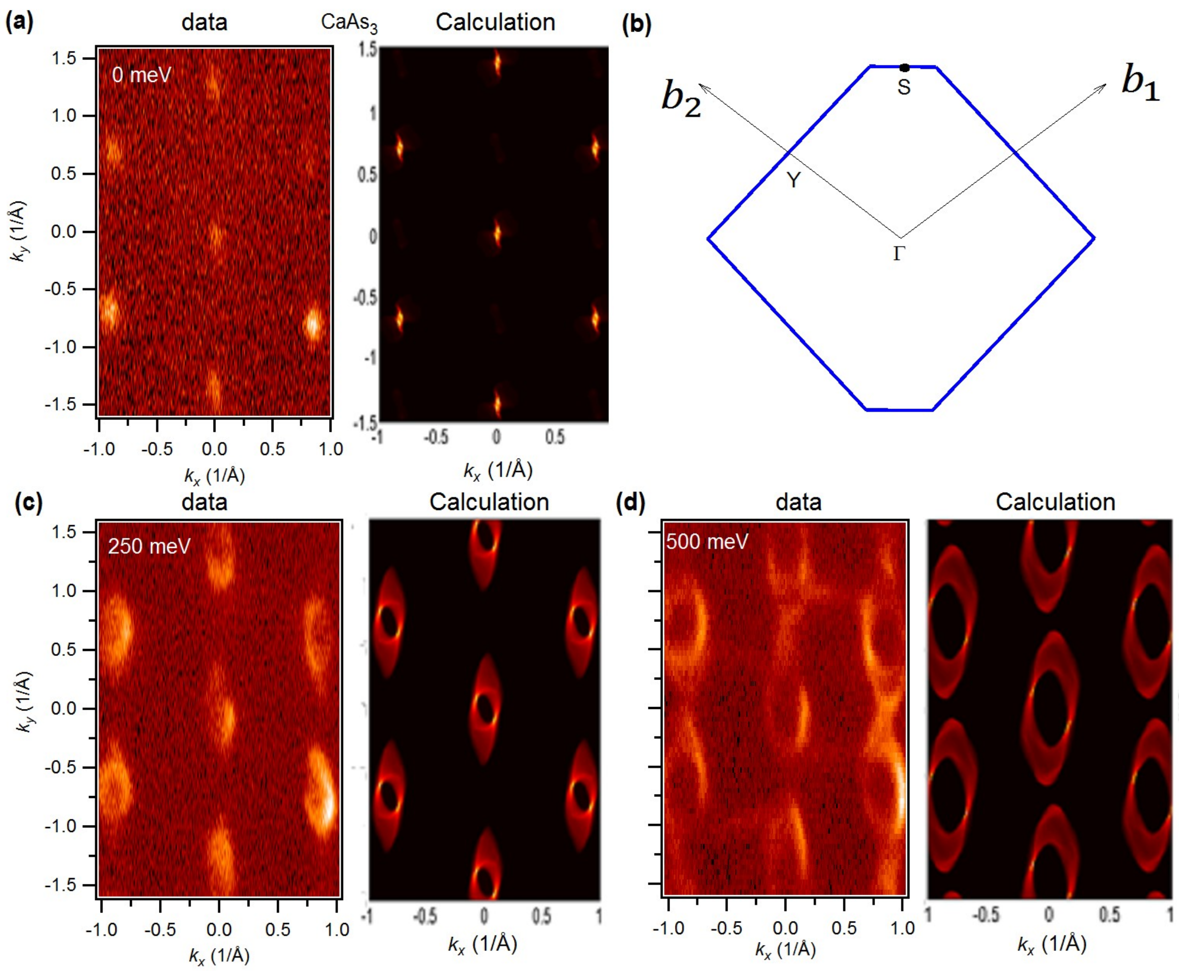

Supplementary figure 4: **Fermi surface map and constant energy contours of $CaAs_3$.** (a) ARPES measured and calculated Fermi surface of $CaAs_3$. Each of the distorted hexagonal represents a separate Brillouin zone where the b-axis is larger than a-axis. (b) Surface Brillouin zone along the (010) plane. (c)-(d) Show the constant energy contour plots at 250 and 500 meV below the chemical potential. All measurements were performed at the HRPES end-station of SLS beamline at a temperature around 18 K.

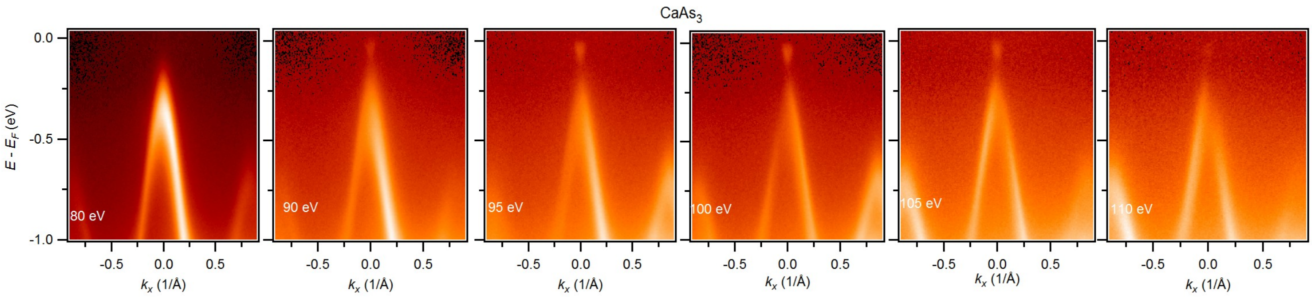

Supplementary figure 5: **Dispersion maps along the Cut1 direction.** (a) Photon energy dependent dispersion maps of $CaAs_3$ along the Cut1 direction. All the experiments were measured at SLS.

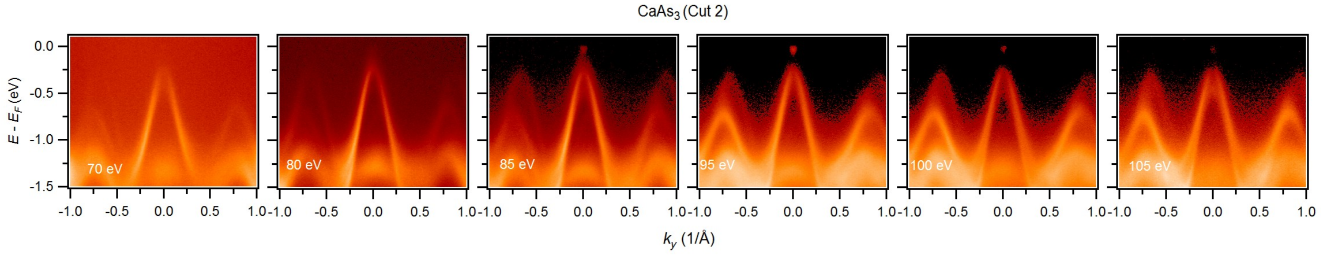

Supplementary figure 6: **Dispersion maps along the Cut2 direction of CaAs**$_3$**.** (a) Photon energy dependent dispersion maps along the Cut2 direction. All the experiments were performed at SLS.

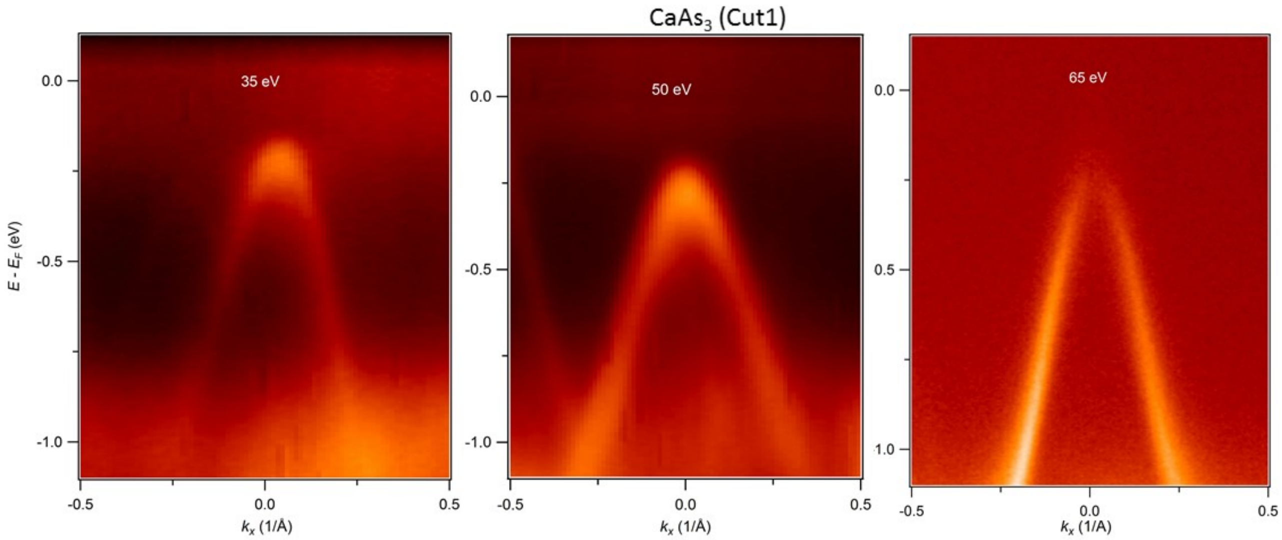

Supplementary figure 7: **Dispersion maps along the Cut1 direction of $CaAs_3$.** Photon energy dependent dispersion maps along the Cut1 direction at relatively lower photon energies. All the experiments were performed at ALS at a temperature around 18 K.

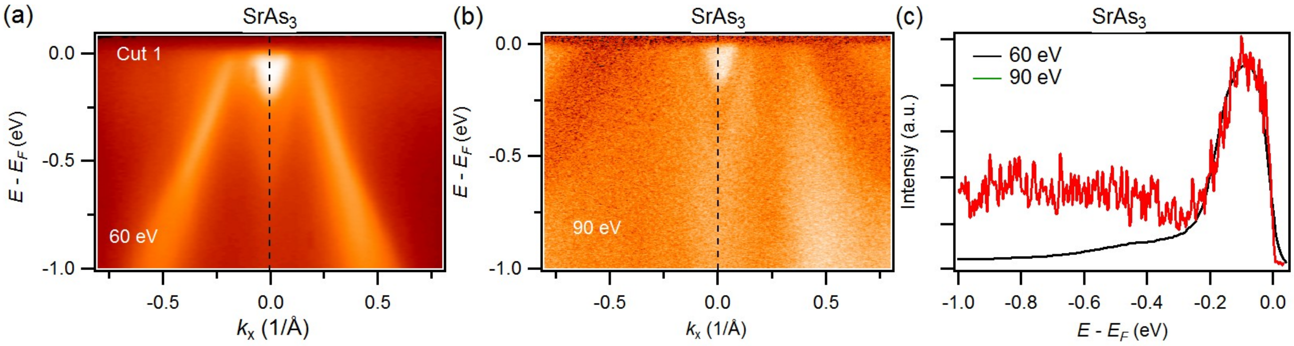

Supplementary figure 8: **Photon-energy-dependent response of surface and bulk state of $SrAs_3$**. (a) Dispersion map along the Cut1 direction measured at ALS. (b) Dispersion map along the Cut1 direction measured at SLS. Photon energies are noted in the plots. (c) Energy distribution curves comparison along the dashed line of 60 eV and 90 eV plots.

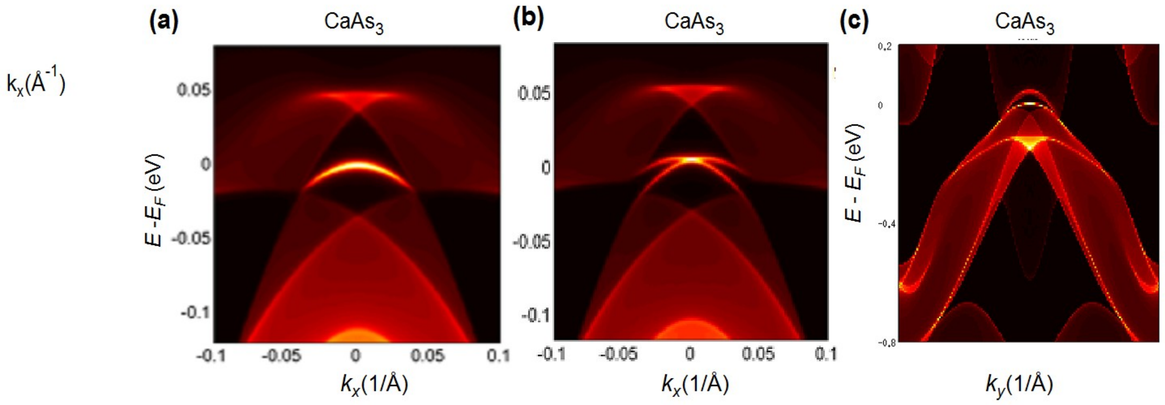

Supplementary figure 9: **Calculated dispersion maps for (010) surface of $CaAs_3$.** (a) Calculated dispersion map along the k_x direction without the inclusion of SOC. (b) Calculated dispersion map along the k_x direction with the inclusion of SOC. (c) Calculated dispersion map along the k_y direction without the inclusion of SOC.

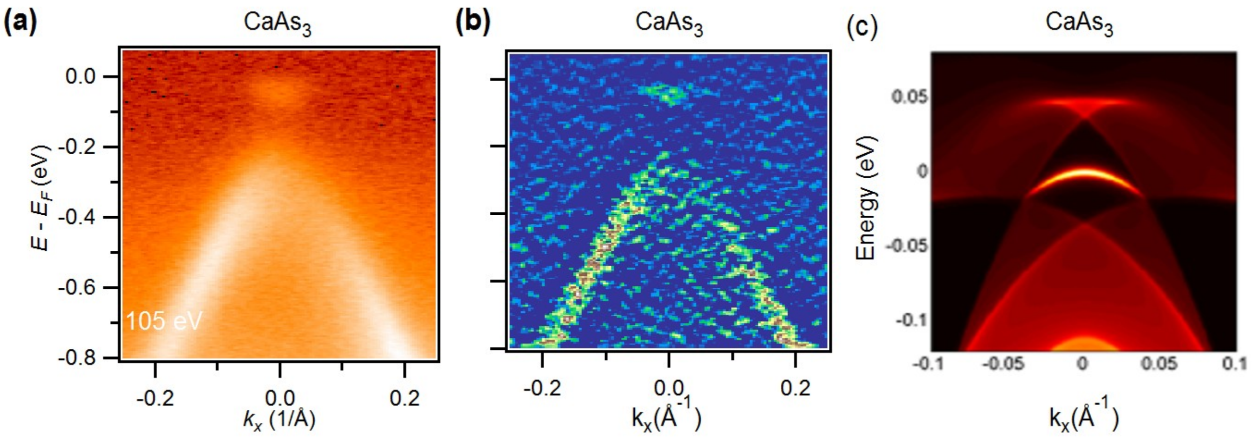

Supplementary figure 10: **Dispersion map along the k_x direction of CaAs$_3$.** (a),(b) Measured dispersion map and second derivative plots using a photon energy of 105 eV, respectively. (c) Calculated dispersion map along the k_x direction with the inclusion of SOC.

Supplementary Note 1: Sample characterization and bulk band calculations.

Supplementary Figures 1(a) and 1(b) show the spectroscopic measured core level measurements of $CaAs_3$ and $SrAs_3$, respectively. Sharp peaks of Ca $3p$ and As $3d$ are observed (see SF. 1(a)). Sharp peak of As $3d$ is only observed for $SrAs_3$ which indicates that the As $3d$ peak is much stronger than the Sr $4s$ peak. Observation of such sharp peak indicates the good surface quality used during our measurements. Supplementary Figures 2(a),(b) represent the calculated bulk band structure of $SrAs_3$, without and with the inclusion of SOC, respectively. Supplementary Figure 2(c) shows the bulk band calculations of $CaAs_3$ without including the SOC. Nodal loops are observed for both system at around the Y point of the Brillouin zone (BZ) in its (010) surface when SOC is excluded. However, for the $CaAs_3$ around 40 meV gap is observed while SOC is included (see SF. 2(d)). Therefore, one can expect to observe a transition from topological nodal-loop (TNL) state to a topological insulator (TI) state in $CaAs_3$. This further supports our main text conclusion that $SrAs_3$ is a nodal-loop semimetal while $CaAs_3$ is gapped out. Moreover, note that the sharp linear dispersive nature of the bands around the Y-points of the BZ in $SrAs_3$, is in contrast to the nearly parabolic nature of the bands in $CaAs_3$.

Supplementary Note 2: Fermi surface evolution of $SrAs_3$ at higher photon energy.

In the main text, we showed that, at the center of BZ, $SrAs_3$ consist of a drumhead surface state. In order to confirm this, we present photon energy dependent Fermi surface maps and dispersion maps along the surface arc. Here, we present more evidences via Fermi surface map and constant energy contour plots at 90 eV incident photon energy. Once again we observe the hexagonal Fermi surface. Most importantly, the bright circular shapes evolve into a point like shape at around 170 meV which is consistent with the 55 eV measured plots and dispersion maps (see main text Fig. 2(a) and 3). Therefore, we can unambiguously conclude that the circular shape is surface originated, hence, provides the first example of the in plane drumhead state in $SrAs_3$.

Supplementary Note 3: Fermi surface and constant energy contour plots of $CaAs_3$

Supplementary Figure 4(a) shows the experimental and calculated Fermi surface maps of $CaAs_3$, within a wide momentum window the surface BZ considered in our calculations is illustrated in Fig. 4(b), where $b_1 = 1.087$ Å^{-1} and $b_2 = 1.077$ Å^{-1}. The agreement between the two data sets is remarkable. In order to figure out the evolution of Fermi contour, we present the constant energy contour plots at about 250 and 500 meV, respectively, below the chemical potential in Fig. 4(c) and Fig. 4(d). In these figures, one can clearly see the distorted hexagonal shape of BZ that is nicely reproduced by our calculations.

Supplementary Note 4: Photon-energy-dependent dispersion maps

In order to determine the nature of the electronic bands associated in the vicinity of the Fermi level, several photon energy dependent energy-momentum dispersion maps were measured over a wide energy window from 80 to 110 eV. We used several photon energies for probing different values of the perpendicular components of the crystal momentum, which enable us to distinguish the surface and bulk origin of the bands. From the results presented in Supplementary Figure 5, it is clear that a single band is crossing in the vicinity of the chemical potential. We

observe notable change in the band dispersion which confirms the band originates from the bulk. Most importantly, the gap near the Fermi level is clearly visible in all photon energy dependent measurements. Particularly, at 80 eV (similarly 110 eV) (see Supplementary Fig. 5) the upper part of the cone completely vanishes which shows an absence of the surface state within the bulk gap in $CaAs_3$. In order to confirm that the upper section of the 80 eV graph does not vanish due to the matrix element effect, we present photon energy dependent dispersion maps along the cut 2 direction (see main text Fig. 4(a)) in Supplementary Fig. 6 and cut 1 direction at relatively lower photon energies in Supplementary Fig. 7. All together they confirm the bulk nature of the band in $CaAs_3$. We have also performed dispersion measurements on $SrAs_3$ under various experimental conditions over a wide range of photon energies to confirm the robustness of the surface state around the Y-point of the BZ. As an example, SF. 8 shows the measured dispersion map at ALS using 60 eV photons (SF.8(a)) and at SLS using a different sample with 90 eV incident photons (SF. 8(b)). Here, the drumhead surface state shows no dispersion,-while bulk bands clearly change with photon energy. A more quantitative proof can be obtained from the comparison of energy distribution curves shown in SF. 8(c).

Supplementary Note 5: Effect of spin orbit coupling and arc length of $CaAs_3$

We have further performed band dispersion calculations, this time by both switching on and off the spin-orbit coupling (SOC) (see Supplementary Fig. 9 (a),(b)). The inclusion of SOC drives the system into a topological insulator phase with a Dirac crossing point at about 40 meV below the chemical potential. The observed large bulk resistivity at low temperature ($\sim 2K$) in our resistivity plot (see main text Fig. 1(d)) further supports this conclusion. To check the Fermi arc size along the various momentum direction, we have performed first-principles calculations along the k_x and k_y (SF. 9) directions for (010) surface (see SF. 9). We have observed a distinct change in the band dispersion for both directions as well as the Fermi arc size which changes by a factor of 4. The estimated Fermi arc length along the k_x direction is about 0.08 Å while it is 0.30 Å along the k_y direction.

Supplementary Note 6: Estimation of the gap size in $CaAs_3$

In order to observe the gap more clearly, we present zoomed view of the dispersion map along the k_x direction and its second derivative plot (see SF. 10). Here, we can clearly see the gap. Therefore, we conclude $CaAs_3$ as topologically trivial material.
